# Effect of Combining Exercise with Adipose-Derived Mesenchymal Stem Cells in Muscle Atrophy Model of Sarcopenia

**DOI:** 10.3390/ijms26020451

**Published:** 2025-01-07

**Authors:** Dong-Hwa Jeong, Min-Jeong Kim, Chul-Hyun Park

**Affiliations:** 1 Department of Physical and Rehabilitation Medicine, Kangbuk Samsung Hospital, Sungkyunkwan University School of Medicine, Seoul 03181, Republic of Korea; ehdghk300@naver.com; 2 Medical Research Institute, Kangbuk Samsung Hospital, Sungkyunkwan University School of Medicine, Seoul 03181, Republic of Korea; 3 Samsung Advanced Institute for Health Sciences & Technology, Sungkyunkwan University, Seoul 06355, Republic of Korea; 4 Research Institute for Future Medicine, Samsung Medical Center, Seoul 06351, Republic of Korea

**Keywords:** sarcopenia, mesenchymal stem cell, exercise, TNF, AMPK

## Abstract

Deterioration in muscle mass, strength, and physical performance due to conditions such as sarcopenia can affect daily activities and quality of life in the elderly. Exercise and mesenchymal stem cells (MSCs) are potential therapies for sarcopenia. This study evaluates the combined effects of exercise and adipose-derived MSCs (ADMSCs) in aged rats with sarcopenia. Eighteen-month-old rats were randomly divided into four groups: control, exercise (Ex), ADMSCs injection (MSC), and ADMSCs injection with exercise (MSC + Ex). Gastrocnemius (GCM) muscle mass increased in the Ex, MSC, and MSC + Ex groups compared to the control group. Although the mean CSA did not differ significantly between the groups, the size distribution of myofibers shifted toward larger sizes in the Ex and MSC + Ex groups. The MSC + Ex group performed best in functional tests, including the rotarod and hot plate tests. The protein expression levels of tumor necrosis factor (TNF) and the p-AMP-activated protein kinase (AMPK)/AMPK ratio in the GCM muscle were the lowest in the MSC + Ex group. This study demonstrates that combining exercise and ADMSC interventions was the most effective treatment for aged sarcopenic rats, suggesting a potential synergistic approach for sarcopenia treatment.

## 1. Introduction

Muscle atrophy is primarily defined as a reduction in muscle mass, whereas sarcopenia is characterized by a progressive decline in muscle mass, muscle strength, and physical function [1,2]. Sarcopenia is categorized into two types. Primary sarcopenia results from age-related factors, while secondary sarcopenia results from other diseases or pathological conditions with or without the involvement of aging [3]. As the global elderly population steadily increases annually, sarcopenia is becoming an increasingly severe concern. Although primarily observed in the elderly, sarcopenia can also affect middle-aged individuals due to various factors [4]. Additionally, recent meta-analyses have identified sarcopenia as a significant predictor of increased all-cause mortality [5]. The etiology of sarcopenia encompasses reduced protein synthesis, chronic inflammation, oxidative stress, impaired function of satellite cells, hormonal deficiencies, nutritional deficiencies, and a lack of physical activity [6,7,8]. These factors cause various diseases and increase multiple risk factors [9], thereby restricting daily activities, causing functional impairments, and diminishing both the quality of life and physical activity levels in the elderly. This highlights the urgent need for treatments to manage sarcopenia [4,10].

Previous studies have demonstrated the benefits of exercise and adequate nutrient intake in managing sarcopenia. Specifically, exercise reduces the incidence of chronic diseases, prevents muscle loss, and extends lifespan, making it an effective strategy for improving the health of the elderly. The impact of exercise on muscle structure and function in sarcopenia is substantial [11,12,13,14]. While research on pharmacological treatments continues to be conducted in an effort to discover a fundamental cure for sarcopenia, an effective pharmacological solution has yet to be established [15,16,17,18]. Consequently, it is crucial to identify treatments that halt the pathological progression of muscle degradation in sarcopenia and enhance muscle regeneration.

Recent studies have shown that mesenchymal stem cells (MSCs) possess therapeutic potential for treating sarcopenia [19]. MSC-based therapies were reported to promote skeletal muscle regeneration in both animal and cellular models [20,21,22,23,24,25]. Among MSCs, adipose-derived MSCs (ADMSCs) offer several advantages: they can be easily obtained in large quantities through minimally invasive procedures and are plentiful in various tissues, making autologous transplantation a viable option. ADMSCs also exhibit exceptional potential to differentiate into various cell types, providing significant prospects for tissue repair and reinforcement [26,27,28]. Research indicates that ADMSCs undergo myogenic differentiation when co-cultured with satellite cells or myoblasts [29,30,31,32,33], with one study demonstrating muscle recovery in a muscle atrophy model [34]. However, the therapeutic effects of ADMSCs in aged rats with sarcopenia have not been definitively established in previous studies.

While individual treatments for sarcopenia often focus on exercise or MSC-based therapies, the combined effects of exercise and MSCs on sarcopenia have not been extensively explored. Therefore, this study examines the effects of exercise only, ADMSC injection only, and the combination of both on the treatment of sarcopenia in an aged rat model.

## 2. Results

### 2.1. Effect of ADMSCs and Exercise on Body and Gastrocnemius (GCM) Muscle Weights in Immobilized Induced Atrophy Models

**Figure 1:**
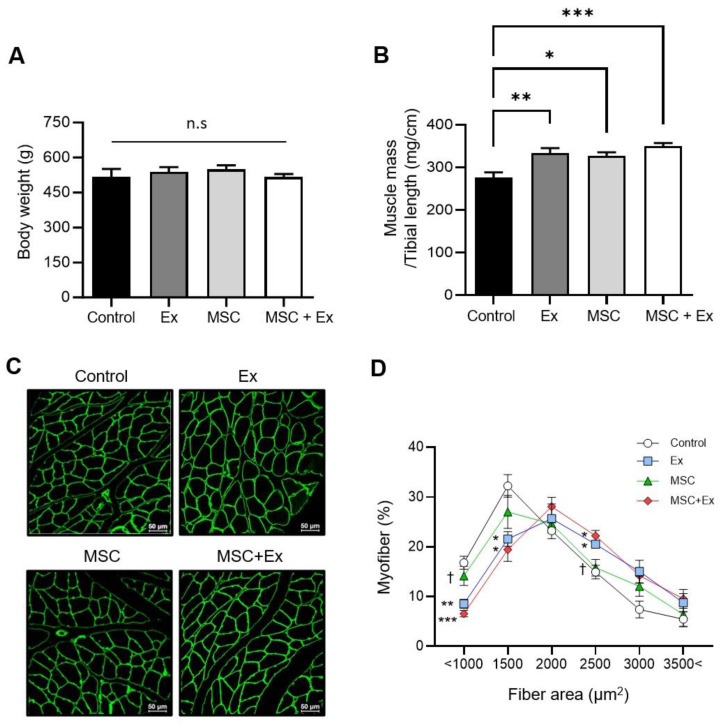
Body weight, muscle weight, and fiber analysis. (**A**) Body weight after the injection of ADMSCs or after exercise on day 28 (*n* = 6, each). (**B**) The ratio of the GCM muscle weight to the tibial length (*n* = 6, each). (**C**) A representative image of anti-laminin-stained GCM muscles. The laminin sections were used for the measurement of the cross-sectional area. (**D**) The frequency distribution of the cross-sectional GCM muscle fiber area (*n* = 5, each). Ex, treadmill exercise; MSC, ADMSC injection; MSC + Ex, ADMSC injection and treadmill exercise group. All data are presented as the mean ± SEM. * *p* < 0.05, ** *p* < 0.01, and *** *p* < 0.001 compared with the control; † *p* < 0.05 compared with the MSC + Ex group.

After 28 d, no differences in body weight were observed among the experimental groups (Figure 1A). However, the GCM muscle weight increased in both the Ex and MSC groups (333.6 ± 11.7 and 326.4 ± 8.8, respectively), with the MSC + Ex group showing a more substantial increase compared to the control (349.2 ± 7.8 vs. 276.5 ± 11.8, respectively) (Figure 1B).

Since we hypothesized that injecting stem cells or engaging in exercise might have a beneficial impact on muscle repair, we measured the myofiber cross-sectional area (CSA) in GCM muscles, and the mean myofiber CSA in GCM muscles did not show statistically significant differences between groups (Supplementary Figure S1). Thus, the distribution of myofiber sizes shifted toward larger sizes in the Ex and MSC + Ex groups compared to the control (Figure 1C,D).

### 2.2. Behavioral Assessments

**Figure 2:**
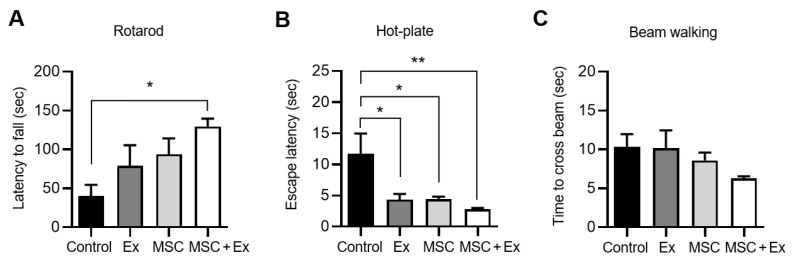
Behavioral changes in each group. (**A**) The retention time (latency to fall) detected by using the rotarod test. (**B**) Response latencies were measured using the hot plate test. (**C**) Walking time (the time to cross the beam) was assessed using the beam walk test. Ex, treadmill exercise; MSC, ADMSC injection; MSC + Ex, ADMSC injection and treadmill exercise group. All data are presented as the mean ± SEM (*n* = 5–6, each). * *p* < 0.05, and ** *p* < 0.01 compared with the control.

To determine whether changes in muscle mass impacted motor and exercise performance, we used the rotarod, hot plate, and beam walk tests. After 28 d, the latency to fall from the rotarod significantly increased in the MSC + Ex group compared to the control (129.6 ± 10.0 vs. 40.1 ± 14.5, respectively), indicating enhanced performance (Figure 2A). Although not statistically significant, the latency to fall also increased in both the Ex and MSC groups (79.0 ± 26.4 and 93.9 ± 20.4, respectively) (Figure 2A). In the hot plate test, the response times to the thermal stimulus decreased in both the Ex and MSC groups, with the fastest response being observed in the MSC + Ex group, indicating improved escape behavior (Figure 2B). In the beam walk test, although the results did not reach statistical significance, a decreasing trend in the time to cross the beam was observed (10.3 ± 1.7 for control, 8.6 ± 1.0 for MSC, and 6.2 ± 0.3 for MSC + Ex) (Figure 2C).

### 2.3. Altered GCM Muscle Inflammatory Cytokine Expression

**Figure 3:**
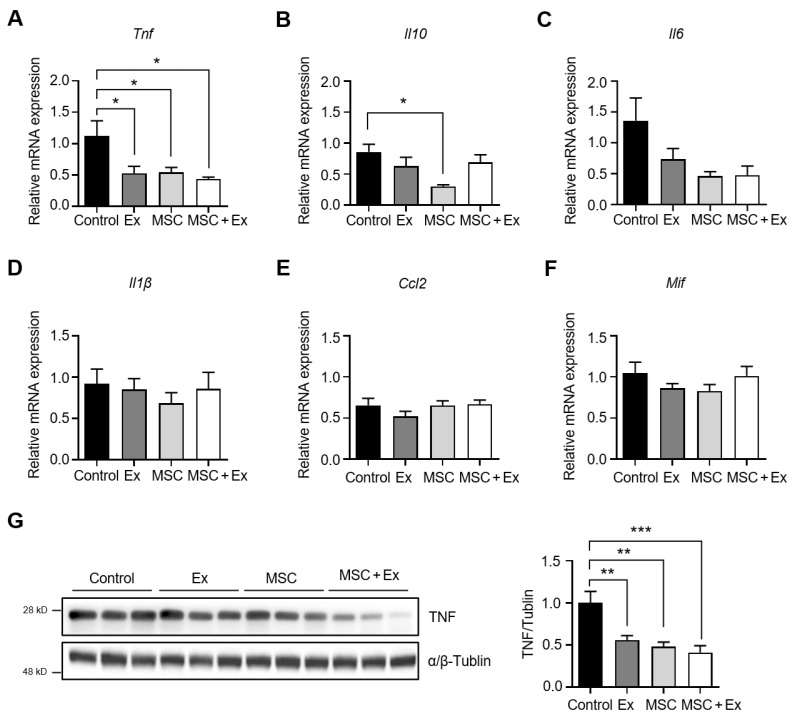
Changes in the level of pro-inflammatory cytokines. The mRNA expression levels of the (**A**) *Tnf*, (**B**) *Il10*, (**C**) *Il6*, (**D**) *Il1β*, (**E**) *Ccl2*, and (**F**) *Mif* genes in the GCM muscle on day 28 (*n* = 5–6, each). (**G**) The relative protein expression of TNF normalized to α/β-TUBLIN (*n* = 6, each). Ex, treadmill exercise; MSC, ADMSC injection; MSC + Ex, ADMSC injection and treadmill exercise group. All data are presented as the mean ± SEM. * *p* < 0.05, ** *p* < 0.01, and *** *p* < 0.001 compared with the control. *Tnf*, tumor necrosis factor; *Il10*, interleukin 10; *Il6*, interleukin 6; *Il1β*, interleukin 1 beta; *Ccl2*, C motif chemokine ligand 2; *Mif*, macrophage migration inhibitory factor.

The mRNA expression levels of six candidate genes (*Tnf*, *Il10*, *Il6*, *Il1β*, *Ccl2*, and *Mif*) of inflammatory markers were measured using Quantitative Reverse Transcription PCR (qPCR) in GCM muscles. The expression levels of *Tnf* were significantly reduced in the Ex, MSC, and MSC + Ex groups compared to the control (0.53 ± 0.11 for Ex, 0.55 ± 0.08 for MSC, and 0.44 ± 0.03 for MSC + Ex vs. 1.13 ± 0.24 for control) (Figure 3A). *Il10* expression was notably lower in the MSC group compared to the control (0.30 ± 0.03 vs. 0.86 ± 0.12, respectively) (Figure 3B). *Il6* expression also showed a decreasing trend in the Ex, MSC, and MSC + Ex groups (0.73 ± 0.17 for Ex, 0.46 ± 0.07 for MSC, and 0.48 ± 0.15 for MSC + Ex vs. 1.36 ± 0.37 for control) (Figure 3C). The expression levels of *Il1β*, *Ccl2*, and *Mif* did not exhibit substantial differences across all groups (Figure 3D–F). Furthermore, TNF protein expression in GCM muscles was diminished in both the Ex and MSC groups (0.56 ± 0.05 for Ex and 0.48 ± 0.05 for MSC), with an even greater reduction observed in the MSC + Ex group compared to the control (0.41 ± 0.08 for MSC + Ex vs. 1.0 ± 0.14 for control; Figure 3G).

### 2.4. Effect of Intramuscular Injection of MSC and Exercise on AMP-Activated Protein Kinase (AMPK) Phosphorylation

**Figure 4:**
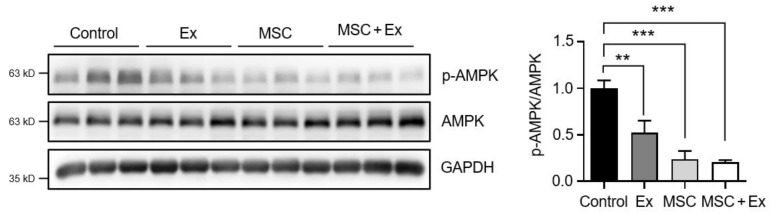
Altered expressions of AMPK phosphorylation. Western blotting was used to analyze expression of AMPK, p-AMPK, and p-AMPK/AMPK in GCM muscles on day 28. Ex, treadmill exercise; MSC, ADMSC injection; MSC + Ex, ADMSC injection and treadmill exercise group. Results are presented as mean ± SEM (*n* = 4–5, each). ** *p* < 0.01, and *** *p* < 0.001 compared with control.

Skeletal muscle regeneration is influenced by the survival and proliferation of MSCs. Previous studies have indicated that AMPK plays a crucial role in regulating the self-renewal and proliferation of stem cells [35,36]. We assessed the protein levels of AMPK after 28 d. Following 2 weeks of exercise and ADMSC injections in rats with immobilization-induced atrophy, a statistically significant decrease in AMPK phosphorylation was observed in the GCM muscles (0.52 ± 0.13 for Ex and 0.24 ± 0.09 for MSC) (Figure 4). Additionally, a further reduction was evident in the MSC + Ex group compared to the control (0.20 ± 0.02 for MSC + Ex vs. 1.0 ± 0.09 for control; Figure 4).

## 3. Discussion

Following the induction of muscle atrophy through hindlimb immobilization and aging, both exercise and ADMSC treatments showed increases in muscle mass and improvements in muscle function. Notably, the combined therapy of exercise and ADMSC injections demonstrated the highest indicators of muscle mass increase and functional enhancement. The combined therapy group exhibited the highest performance in the rotarod test and the fastest escape in the hot plate test. Furthermore, the combined therapy group presented the lowest TNF level and activated AMPK, which were possibly related to the pathogenesis of sarcopenia. A previous similar study demonstrated that in rats with muscle atrophy induced by spinal cord injury, the combination of exercise and bone marrow stromal cell transplantation had the potential to accelerate protein synthesis and hypertrophy [37]. This was a denervation-induced muscle atrophy model. However, immobilization-induced muscle atrophy involves a physiologically different mechanism for decreasing muscle mass compared to nerve injuries like spinal cord injury. The present study reports that the combined therapy of exercise and ADMSC injections was the most efficacious and synergistic intervention for the model of muscle atrophy induced by immobilization and aging.

Muscle atrophy-induced sarcopenia is characterized by declines in muscle mass and function. In our study, the Ex, MSC, and MSC + Ex groups showed an increase in the GCM muscle mass compared to the control. Although statistical significance was not achieved, the distribution of the CSA in the GCM muscle indicates that the Ex and MSC + Ex groups had a higher proportion of fibers of larger size compared to the control group. These findings align with recent studies demonstrating that MSCs and exercise therapy increase muscle mass and the CSA. Wang et al. [34] demonstrated that the intravenous injection of ADMSCs in dexamethasone-induced sarcopenic rats improved body lean mass, muscle mass, and the CSA of the GCM muscle. Another recent study showed that in sarcopenic mice, the injection of umbilical cord-derived MSCs either via the tail vein or through local intramuscular administration resulted in improved muscle mass, CSA of the GCM muscle, and overall muscle strength [24]. Herein, we conducted a muscle function assessment using the rotarod and hot plate tests, revealing that the Ex, MSC, and MSC + Ex groups exhibited enhanced balance and rapid escape performance compared to the control group. Notably, the MSC + Ex group demonstrated the most significant improvements. Therefore, using ADMSCs in conjunction with exercise interventions effectively improved both muscle mass and function.

TNF is known to play a significant role in the pathogenesis of sarcopenia [38,39,40]. Previous studies have reported that TNF promotes inflammatory responses and increases protein degradation in skeletal muscle, decreasing muscle mass and impairing function. Therefore, the decreased levels of TNF, a representative pro-inflammatory cytokine, in the Ex, MSC, and MSC + Ex groups compared to the control indicate that exercise and ADMSC injections suppress inflammation, thereby facilitating the mitigation of sarcopenia. Previous animal model studies support our current results, showing that treadmill exercise often leads to decreased TNF levels [41,42,43]. Additionally, a recent study demonstrated that the local injection of ADMSCs into the midpoint of the GCM muscle induced reductions in TNF and IL-6 levels in dystrophin-deficient mice [44]. In the present study, we observed that the TNF levels were the lowest in the MSC + Ex group, indicating a synergistic effect of the combination of exercise and ADMSCs in decreasing inflammation associated with sarcopenia.

AMPK regulates both protein synthesis and degradation in muscle growth and is involved in various processes, such as muscle size in a basal state, muscle hypertrophy, muscle atrophy, and muscle regeneration [45]. Bolster et al. demonstrated that subcutaneous injection of the AMPK activator 5-aminoimidazole-4-carboxamide ribonucleoside (AICAR) in rats resulted in a 45% reduction in protein synthesis rates in skeletal muscle compared to the control group [46]. However, the role of AMPK in muscle atrophy remains uncertain. Studies on rodent skeletal muscle subjected to 1–4 weeks of hindlimb unloading have reported conflicting results: some reported an increase in AMPK phosphorylation [47], whereas others observed a decrease [48,49,50]. Additionally, other research has found no effect of hindlimb unloading on AMPK activity or the phosphorylation of acetyl-CoA carboxylase, a target and biomarker of AMPK [51]. Furthermore, previous studies have shown that activated AMPK plays a crucial role in reducing the self-renewal and proliferation of MSCs in vitro and ex vivo [35,36]. This supports the findings of the present study, where the p-AMPK/AMPK ratio was decreased in the Ex, MSC, and MSC + Ex groups compared to the control. These results suggest that a reduction in AMPK phosphorylation at Thr-172 may lead to increased muscle mass and enhanced MSC self-renewal and proliferation, potentially alleviating sarcopenia.

However, there are several limitations to consider. First, the present study did not include muscle strength as an outcome measure. Although grip strength is an important marker of sarcopenia, its validity in rats was unsatisfactory as the strength values varied among individual rats during our pilot study. Second, muscle atrophy was induced through immobilization and aging to establish a sarcopenia model. Even though there are previous reports that showed this model being used as a sarcopenic rat model [52,53,54,55,56,57], this approach may not fully demonstrate a true sarcopenia model, highlighting the need for a more appropriate and refined model representing primary sarcopenia. Third, ultrasound-guided intramuscular injection was performed to ensure that ADMSCs were accurately injected into the targeted regions of the gastrocnemius muscle. However, tracking their homing and visualizing them post-injection proved challenging, indicating the need for additional strategies, such as cell staining or labeling. Fourth, the dosage of MSCs was not determined. We based our MSC transplant design on a previous study [58], but further research is needed to determine the optimal concentration of MSCs for injection according to age and to identify potential side effects. Moreover, optimizing variables such as the route of administration, timing, and cell condition that affect MSCs’ survival and homing is crucial for maximizing therapeutic efficacy [59]. Fifth, it is necessary to assess the most effective exercise regimen when combining MSC injection with exercise. Sixth, while we employed immunohistochemistry to assess the cross-sectional area (CSA) of myofibers, myonuclear staining was not included in this study. According to previous studies [60], myonuclear staining is not deemed essential for CSA analysis. However, incorporating myonuclear staining in future investigations could provide additional insights into myofiber structure. Finally, this study was conducted with an evaluation period of only 28 days, which is relatively short. A follow-up study is necessary to assess the long-term effects.

## 4. Materials and Methods

### 4.1. Animals and Muscle Tissue Preparation

For this muscle atrophy-induced sarcopenia study, experiments were conducted on 18-month-old male rats (DBL, Chungcheongbuk-do, Republic of Korea). The rats were randomly divided into four groups, each comprising six rats: control (control), treadmill exercise (Ex), ADMSC injection (MSC), and ADMSC injection with treadmill (MSC + Ex) groups. After the experiments, the rats were anesthetized with 5% isoflurane and euthanized in a carbon dioxide chamber, and GCM muscle samples were collected. The GCM muscles from both hindlimbs were excised and weighed. One hindlimb was immediately frozen in liquid nitrogen and stored at −80 °C, while the other was used to create a formalin-fixed, paraffin-embedded block for a muscle histology analysis. All animal experiments received approval from the Kangbuk Samsung Hospital Research Institute’s Animal Care Committee (Approval ID: 202011133; approval date: 12 October 2020).

### 4.2. Velcro Immobilization

**Figure 5:**
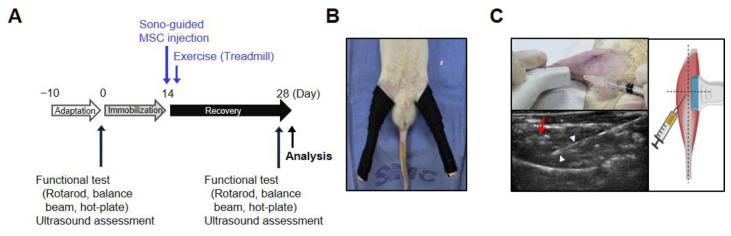
The experimental design for the immobilization-induced atrophy model. (**A**) A schematic of the experimental schedule of rats in this experiment. (**B**) A rat’s hindlimb was fixed with adhesive tape and Velcro bandage. (**C**) Ultrasound images of the intramuscular injection of ADMSCs into the GCM muscle (**left**). The red arrow indicates injected ADMSCs, and the white arrowhead shows the syringe needle. The intersection of the dotted lines represents the target point of injection, indicating the analyzed region of the GCM (**right**).

The hindlimb Velcro immobilization method was adopted to induce a muscle atrophy model [61,62]. The procedure was conducted under anesthesia with 2% isoflurane. The rats’ hindlimbs and surrounding fur were shaved. Each hindlimb was kept in an extended position to maintain the knee and ankle joints in a stretched state, secured with surgical and Velcro tapes around the limb, and then reinforced with adhesive plaster. In the initial stage of the experiment, all groups of rats were subjected to Velcro immobilization for 14 d, during which they were able to freely move within the cage using their forelimbs and had access to food and water. After the Velcro was removed, the treadmill exercises, ADMSC injections, and combined treatments were initiated to enhance muscle regeneration and promote functional improvement in the muscles (Figure 5A).

### 4.3. ADMSC Preparation and Ultrasound-Guided Intramuscular Injection

ADMSCs at passage 1, which were isolated from interscapular brown fat tissue of rats, were obtained from iXCells Biotechnologies (10RA-002, San Diego, CA, USA). The cells were cultured in adipose-derived stem cell growth medium (MD-0003, iXCells Biotechnologies, San Diego, CA, USA), which includes 500 mL of Basal Medium, 50 mL of FBS, 1 mL of adipose-derived stem cell growth supplement, and 5 mL of antibiotic-antimycotic (100X), in a humidified 5% CO_2_ incubator at 37 °C according to the manufacturer’s protocol. ADMSCs were assessed based on their passage number, morphology, and stem cell potency with reference to previous studies [63,64]. The purchased cells (at passage 1) were counted and seeded to 5 × 10^3^ cell/cm^2^. When the cells reached 70–80% confluency, they were sub-cultured at a density of 5 × 10^3^ cells/cm^2^. Flow cytometry analysis was conducted on cells at passage 2 to confirm stem cell identity before creating a stock, and they tested positive for stem cell marker CD90 and negative for CD18 and CD45 (Supplementary Figure S2). During the immobilization period, frozen cells were thawed and allowed to recover. Once they reached passage 3, the cells were dissociated into single cells and stained with trypan blue, and only the viable cells were used for the experiments. Following the removal of the Velcro, rats in both the MSC and MSC + Ex groups received injections of 1.0 × 10^6^ cells in Phosphate-Buffered Saline (PBS) into their right and left GCM muscles, respectively. Meanwhile, the control and Ex groups were injected with the same volume of PBS as the ADMSC injection groups [58,65]. The injections were performed under ultrasound guidance to ensure accurate delivery into the GCM muscle, specifically targeting the intersection point of the *x*-axis and *y*-axis at the thickest portions of the muscle.

### 4.4. Ultrasound Assessment

Ultrasound evaluations of the GCM muscle were performed in triplicate: prior to Velcro immobilization, during the ultrasound-guided injection of ADMSCs and PBS (Figure 5B), and following the recovery period. The measurements taken included the distance (mm), circumference (cm), and CSA (cm^2^) of the GCM muscle under ultrasound guidance (Supplementary Table S1).

### 4.5. Exercise Intervention

To explore the relationship between ADMSC injection and exercise during the recovery period in rats with muscle atrophy, treadmill exercise was performed (DJ-344, Daejonglab, Seoul, Republic of Korea) [66,67,68]. Rats in the Ex and MSC + Ex groups underwent a 3 d adaptation period before Velcro immobilization. On day 1, rats adapted to the treadmill for 20 min. On day 2, they exercised at a speed of 10 m/min for 20 min, and on day 3, the duration was extended to 40 min at the same speed. Following Velcro removal, during the recovery period, the rats engaged in training sessions of 60 min at 10 m/min, 5 d a week, for 2 weeks.

### 4.6. Functional Tests

Functional tests, including the rotarod, beam walk, and hot plate tests, were conducted on days 0 and 28 of the experimental period. The rotarod test (DJ-345, Daejonglab, Seoul, Republic of Korea) assessed muscle function by gradually increasing the speed from 4 to 40 rpm over 300 s, with three trials being conducted with 15 min intervals between each. The average value was recorded.

The beam walk assessed motor balance and coordination by timing how long it took each rat to traverse a beam measuring 60 mm in width and 120 cm in length [69]. The journey from the starting point to a dark box (25 × 25 × 25 cm) at the opposite end was timed three times and averaged. The hot plate test (JD-A-10A, Jeongdo Bio, Seoul, Republic of Korea) evaluated sensory and motor functions by measuring the time taken for specific responses to occur, such as climbing, jumping, stamping, and licking of the forelimbs and hindlimbs [70]. Rats were placed on a hot plate maintained at a temperature in the range of 50–55 °C, and the reaction times were recorded.

### 4.7. Immunohistochemistry

The following antibodies and reagents were used to determine fiber size: (1) rabbit polyclonal anti-laminin primary antibody (L9393, Sigma-Aldrich, St. Louis, MO, USA); (2) donkey anti-rabbit IgG (H + L) secondary antibody, Alexa Fluor488 (A-21206, Thermo Fisher Scientific, Waltham, MA, USA); (3) vibrance antifade mounting medium with DAPI (H-1800, Vector Laboratories, Newark, CA, USA); and (4) pepsin (P6887, Sigma-Aldrich, St. Louis, MO, USA).

The GCM muscles were fixed in 10% buffered formalin for 48 h and then embedded in paraffin blocks. Prior to staining, slides were prepared from the central region of the GCM injection site and sectioned at a thickness of six micrometers. The slides were deparaffinized, rehydrated, and incubated for 1 h with pepsin (4 mg/mL in 0.01 M HCl, pH approximately 2.0) at 37 °C in a humidified chamber for antigen retrieval. Afterward, the slides were incubated in a blocking solution (5% Bovine Serum Albumin with 0.1% Triton X-100 in PBS) for 1 h at room temperature (22–24 °C). Following blocking, the sections were incubated overnight with laminin antibody (diluted 1:25) at 4 °C. The slides were then washed three times with PBS for 10 min each, and secondary antibodies (diluted 1:1000) were applied for 1 h at 37 °C in a humidified chamber. Following this, the slides were washed three times with PBS and mounted with 4′,6-diamidino-2-phenylindole-containing medium. The slides were left to dry at room temperature or at 4 °C. Five sections from the central region of each stained slide were randomly selected and imaged at 20× magnification using a confocal microscope (STELLARIS 5, Leica Microsystems, Deerfield, IL, USA) with Leica LAS X imaging software version 3.7.6.25997. The CSA was analyzed using ImageJ software version 1.54.

### 4.8. RNA Extraction and Quantitative Reverse Transcription PCR

Total RNA was isolated using TRIzol Reagent (Thermo Fisher Scientific, Waltham, MA, USA). mRNA was reverse-transcribed using the High-Capacity RNA-to-cDNA Kit (Thermo Fisher Scientific, Waltham, MA, USA). qPCR was performed using specific primers and SYBR Green on an LC480 instrument (Roche, Welwyn Garden City, UK). A list of the primers used is provided in Supplementary Table S2. The relative mRNA expression levels were analyzed using the 2^−ΔΔCt^ method with *GAPDH* serving as the reference gene.

### 4.9. Western Blotting

Total protein from GCM muscle tissues was homogenized using RIPA lysis buffer (Thermo Fisher Scientific) containing a protease inhibitor, followed by centrifugation at 13,500× *g* at 4 °C for 15 min to extract the proteins. The proteins were then separated on Sodium Dodecyl Sulfate–polyacrylamide gels and transferred for immunoblotting using specific antibodies, including GAPDH (#2118, Danvers, MA, USA), α/β-Tubulin (#2148, Danvers, MA, USA), AMPK (#5831, Danvers, MA, USA), phosphorylated AMPK (#2535, Danvers, MA, USA), anti-rabbit IgG HRP-linked antibody (#7074, Danvers, MA, USA), anti-mouse IgG HRP-linked antibody (#7076, Cell Signaling Technology, Danvers, MA, USA), and TNF-α (#SC-52746, Santa Cruz, Dallas, TX, USA). Primary antibodies were diluted to a ratio of 1:1000 and secondary antibodies to a ratio of 1:10,000. Protein detection was conducted using Enhanced Chemiluminescence solution and imaged with a ChemiDoc system and then quantified using FUSION FX software version 18.10.

### 4.10. Statistical Analysis

After data collection, statistical analysis was performed using GraphPad Prism version 10.0.3. For comparisons between groups subjected to different treatments such as behavioral tests, qPCR, and Western blotting, a one-way analysis of variance (ANOVA) was conducted, followed by Tukey’s multiple comparisons test. For analyses of CSA differences between groups, a two-way ANOVA was utilized, also followed by Tukey’s multiple comparisons test. All data are presented as the mean ± standard error of the mean. Statistical significance was established at a *p*-value < 0.05.

## 5. Conclusions

In conclusion, this study demonstrates that the combination of exercise plus MSC injection significantly enhances muscle mass and function in a model of muscle atrophy induced by immobilization and aging. This finding is supported by the observed reduction in TNF levels and the activation of AMPK. Thus, these findings provide a foundation for further clinical trials investigating the use of MSCs as a treatment for sarcopenia. In the long term, they may represent a potential therapeutic approach for patients suffering from musculoskeletal diseases.

## Data Availability

All data are presented within the manuscript and Supplementary Materials.

## Supplementary Materials

The following supporting information can be downloaded at: https://www.mdpi.com/article/10.3390/ijms26020451/s1.
