# Supplementary material for: Effect of Combining Exercise with Adipose-Derived Mesenchymal Stem Cells in Muscle Atrophy Model of Sarcopenia
Source: Int J Mol Sci. 2025 Jan 7;26(2):451. doi: 10.3390/ijms26020451 (PMC11764817; doi:10.3390/ijms26020451)
Supplement: Supplementary file 1 [file ijms-26-00451-s001.zip › ijms-3353931-supplementary.pdf]

Supplementary Material

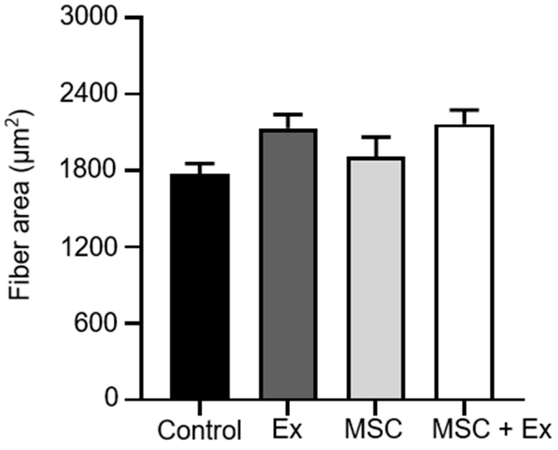

Figure S1. Myofiber CSA in GCM muscles. Ex, treadmill exercise; MSC, ADMSCs injection; MSC + Ex, ADMSCs injection and treadmill exercise group. All data are presented as mean ± standard error of the mean.

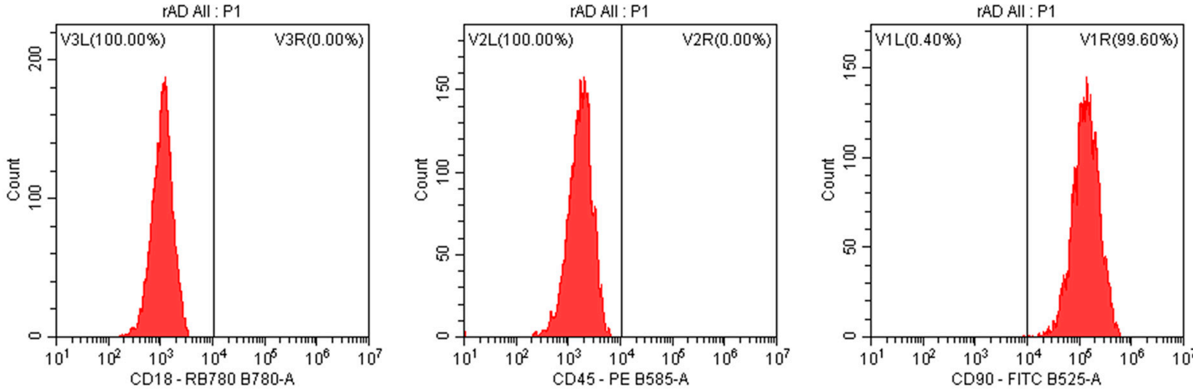

Figure S2. Flow cytometric analysis of ADMSCs. ADMSCs showed CD18 and CD45 negative and CD90 positive features.

Table S1. Measurement of GCM muscles using ultrasound technique

Measurement	Group	Day 0	Day 28
Distance(mm)	Control	7.12 ± 0.33	7.43 ± 0.34
	Ex	7.23 ± 0.40	7.49 ± 0.23
	MSC	7.10 ± 0.16	7.50 ± 0.31
	MSC + Ex	7.21 ± 0.35	7.81 ± 0.34
Circumference(cm)	Control	7.24 ± 0.37	7.31 ± 0.14
	Ex	7.16 ± 0.36	7.29 ± 0.29
	MSC	7.09 ± 0.39	7.35 ± 0.18
	MSC + Ex	7.35 ± 0.28	7.54 ± 0.19
Area(cm^2)	Control	1.73 ± 0.19	1.80 ± 0.07
	Ex	1.76 ± 0.19	1.79 ± 0.07
	MSC	1.69 ± 0.13	1.87 ± 0.08
	MSC + Ex	1.81 ± 0.20	1.92 ± 0.11

Table S2. Quantitative reverse transcription-PCR primer sequences

Gene	Sequence	Product size (bp)	Accession no.
Tnf	F - TAGCCCACGTCGTAGCAAAC<br>R - GCAGCCTTGTCCCTTGAAGA	170	NM_012675.3
Il10	F - GGTTGCCAAGCCTTATCGGA<br>R - TCAGCTTCTCACCCAGGGAA	115	NM_012854.2
Il1β	F - GAAATGCCACCTTTTGACAGTG<br>R - CTGCCACAGCTTCTCCACAG	198	NM_031512.2
Il6	F - GACAAAGCCAGAGTCCTTCAGA<br>R - AGGAGAGCATTGGAAATTGGGG	113	NM_012589.2
Ccl2	F - CTGTCATGCTTCTGGGCCTG<br>R - TCTCCAGCCTACTCATTGGGA	138	NM_031530.1
Mif	F - GTGACCACCAATGTTCCCCG<br>R - GTGCACTGCGATGTACTGTG	108	NM_031051.2
Gapdh	F - TCACCACCATGGAGAAGGC<br>R - GCTAAGCAGTTGGTGGTGCA	169	NM_017008.4

Ccl2, C motif chemokine ligand 2; *Gapdh*, glyceraldehyde 3-phosphate dehydrogenase; *Il10*, interleukin 10; *Il6*, interleukin 6; *Il1β*, interleukin 1 beta; *Mif*, macrophage migration inhibitory factor; *Tnf*, tumour necrosis factor.
